# Piceatannol and Other Wine Stilbenes: A Pool of Inhibitors against α-Synuclein Aggregation and Cytotoxicity

**DOI:** 10.3390/nu8060367

**Published:** 2016-06-15

**Authors:** Hamza Temsamani, Stéphanie Krisa, Marion Decossas-Mendoza, Olivier Lambert, Jean-Michel Mérillon, Tristan Richard

**Affiliations:** 1 Université de Bordeaux, ISVV, EA 4577 Oenologie, Faculté de Pharmacie, MIB (GESVAB), Villenave d’Ornon 33882, France; temsamani.hamza.ht@gmail.com (H.T.); Stephanie.Krisa@u-bordeaux.fr (S.K.); jean-michel.merillon@u-bordeaux.fr (J.-M.M.); 2 INRA, ISVV, USC 1366 Oenologie, Villenave d’Ornon 33882, France; 3 CBMN-UMR 5248 CNRS, Université de Bordeaux, IPB, Allée Geoffroy St. Hilaire, Pessac 33600, France; m.decossas@cbmn.u-bordeaux.fr (M.D.-M.); o.lambert@cbmn.u-bordeaux.fr (O.L.)

**Keywords:** stilbene, piceatannol, Parkinson’s disease, α-synuclein

## Abstract

The aggregation of α-synuclein is one on the key pathogenic events in Parkinson’s disease. In the present study, we investigated the inhibitory capacities of stilbenes against α-synuclein aggregation and toxicity. Thioflavin T fluorescence, transmission electronic microscopy, and SDS-PAGE analysis were performed to investigate the inhibitory effects of three stilbenes against α-synuclein aggregation: piceatannol, ampelopsin A, and isohopeaphenol. Lipid vesicle permeabilization assays were performed to screen stilbenes for protection against membrane damage induced by aggregated α-synuclein. The viability of PC12 cells was examined using an MTT assay to assess the preventive effects of stilbenes against α-synuclein-induced toxicity. Piceatannol inhibited the formation of α synuclein fibrils and was able to destabilize preformed filaments. It seems to induce the formation of small soluble complexes protecting membranes against α-synuclein-induced damage. Finally, piceatannol protected cells against α-synuclein-induced toxicity. The oligomers tested (ampelopsin A and hopeaphenol) were less active.

## 1. Introduction

Parkinson’s disease (PD) is the second most encountered neurodegenerative disorder after Alzheimer’s disease [1]. PD is characterized by the loss of the dopaminergic neurons in the substantia nigra of patients. One of the major hallmarks of PD and some other related disorders is the presence of intracellular inclusions known as Lewy bodies that develop inside nerve cells. They are mainly constituted of α-synuclein fibrils [2,3]. α-synuclein is a 140-residue protein abundantly expressed in brain, where it is concentrated in presynaptic nerve terminals [4]. Convergent genetic, biochemical, and animal studies indicate that the accumulation and aggregation of α-synuclein protein play a fundamental role in the etiology and pathogenesis of PD and related disorders [5,6]. The protein aggregation follows a pathway from monomers to protofibrils and fibrils. The role of these different physical forms is still controversial. While deposits of α-synuclein fibrils in Lewy bodies are a ubiquitous pathological feature of PD [2], growing evidence has shown that the most toxic species are the soluble α-synuclein oligomeric intermediates [7,8]. Particularly, these species could target biological membranes, possibly forming structures with pore-like morphologies that may induce toxicity by the disruption of the cellular membranes [9,10].

Characterizing new small compounds that are able to inhibit α-synuclein aggregation and to lead to non-toxic aggregated species is therefore one of the paradigms of interest in the prevention of PD [11,12]. Consequently, intensive research is aimed at identifying small organic molecules that can inhibit and/or disaggregate α-synuclein aggregate. Many studies have focused on phenolic compounds, indicating that some of them can have a strong inhibitory effect, leading to the stabilization of non-toxic oligomer species [13,14,15,16,17,18]. Some of these molecules were found to strongly protect against membrane perturbation induced by aggregated α-synuclein [19]. Among the polyphenol classes, stilbenes have been shown to possess a large panel of health-related beneficial effects [20,21]. The stilbene structure derives from resveratrol with an essential skeleton constituted by two aromatic rings joined by an ethylene bridge (C6–C2–C6). These compounds were first identified in grapes, but their abundance in nature has since been established, and new dietary sources are still being identified [22]. We recently reported that stilbenes inhibit β-amyloid fibril formation [23]. Our findings suggest the formation of non-toxic soluble complexes between polyphenol and β-amyloid [24,25].

In this study, we investigated the effects of three stilbenes extracted from vine stalks: a monomer (piceatannol), a dimer (ampelopsin A), and a tetramer (isohopeaphenol). Aggregation inhibitors were identified with thioflavin T (thT) fluorescence assays along with their fibril destabilizing propensity. Transmission electron microscopy (TEM) and SDS-PAGE analysis were performed to correlate fluorescence measurements with direct observations of the state of fibrillation. Finally, in order to determine whether stilbenes lead to the formation of non-toxic species, their protective effects against α-synuclein-induced membrane permeabilization and cytotoxicity on neuronal PC12 cells were investigated.

## 2. Materials and Methods

### 2.1. Synthetic Peptides and Polyphenols

Purified recombinant human α-synuclein was purchased from Alexotech AB (Umeå, Sweden) and was used without further purification. Solutions of 140 µM of α-synuclein were prepared in a 20-mM Na_2_HPO_4_, 140-mM NaCl buffer at pH 7.4 and sonicated for 2 min prior to each experiment. Piceatannol, ampelopsin A, and isohopeaphenol were isolated from *Vitis vinifera* vine stalks [26]. Purity was controlled by HPLC measurements. The stilbenes were kept as 20-mM stock solutions in dimethylsulfoxide (DMSO).

### 2.2. α-Synuclein Fibril-Inhibiting Assay

For fluorescence measurements, thT was used at a final concentration of 20 µM. α-synuclein (70 µM final concentration) was incubated in a 96-well plate in the presence or absence of stilbenes (100 and 200 µM, final concentration). The plate was incubated at 37 °C for 0–4 days with agitation (300 rpm). Fluorescence emission was measured with a Fluostar Optima plate reader (BMG Labtech, Offenburg, Germany) set at 450 nm for excitation and 485 nm for emission. Blanks of each compound were subtracted from the measured fluorescence. Each condition was triplicated.

### 2.3. α-Synuclein Fibril Destabilizing Assay

α-synuclein (70 µM final concentration) was incubated in a 96-well plate. After 4 days of aggregation, polyphenols were added at final concentrations of 100 and 200 µM. Fluorescence emission was recorded for 2 h as described above.

### 2.4. Fibril Observation by Transmission Electron Microscopy (TEM)

Aliquots of each sample were deposited for 2 min on carbon-coated copper grids submitted to a glow discharge (0.3 mBar, 2 A). After quick washing in ultrapure water, negative staining using 4% uranyl acetate for 2 min was then performed. Observations were made with a CM120 transmission electron microscope (FEI, Hillsboro, OR, USA) using 2 k × 2 k USC1000 slow-scan CCD camera (Gatan, Pleasanton, CA, USA).

### 2.5. Gel Electrophoresis

SDS-PAGE was carried out according to Meng *et al*. [27]. Synuclein (70 µM) was incubated with thT at 20 µM with or without stilbenes (100 and 200 µM). After 6 days, samples were centrifuged at 14,000 rpm to separate the insoluble aggregates in the pellet from the soluble ones in the supernatant. The pellet was resuspended in 15 µL of phosphate buffer. Five microliters of charge buffer (0.25 M Tris, 8% SDS, 60% glycerol, 0.08% bromophenol blue, pH 6.8) were added to both the supernatant and the pellet. The samples were then heated at 50 °C for 3 min and loaded on 10%–20% Tris-Tricine gels from BioRad. The migration buffer was 0.1 M tricine, 0.1 M Tris, 0.55% SDS, and pH 8.1, and migration was performed with a Mini-PROTEAN Tetra Cell from Biorad. Gels were then stained with Coomassie Blue (0.1% Coomassie R250, 10% acetic acid, 40% methanol).

### 2.6. Calcein Leakage Assay

Phosphatidyl inositol was purchased from Avantis Polar Lipids and used without further purification. The lipid was dissolved in a chloroform solution of 10 mg/mL. To prepare large unilamellar vesicles (LUVs), a thin lipid film was formed by drying the lipid in a glass tube using a gentle nitrogen stream. The glass tube was then placed in a vacuum for 4 h in order to remove any remaining solvent. Calcein was purchased from Sigma-Aldrich and prepared in a 70 mM final solution of 10 mM Hepes, 150 mM NaCl, and 1 mM EDTA at pH 7.4. This solution (1 mL) was added to the dry lipidic film and then submitted to 10 freeze-thaw cycles in liquid nitrogen and 40 °C water. It was then extruded with an Avantis polar lipid Mini-Extruder using sequentially a 1-µm, 0.5-µm, and 0.1-µm filter. Liposomes were purified through a Sephadex G-75 size exclusion column (Sigma-Aldrich, Lyon, France), and their concentration was estimated by phospholipid quantification according to Rouser *et al*. [28]. Samples of α-synuclein (70 µM) with or without stilbenes (100 and 200 µM) were incubated for 1 week in a 96-well plate. For fluorescence measurements, the LUV final concentration was 20 µM. Aggregated α-synuclein samples were diluted sevenfold after their addition to a LUV-containing sample. Fluorescence signal was recorded at 520 nm after excitation at 490 nm with excitation and an emission slit of 5 nm (Varian Cary Eclipse fluorescence spectrophotometer). At the end of the calcein leak triggered by α-synuclein, Triton X-100 was added to the media to release all the calcein from the LUVs. Measurement points were plotted against the fluorescence signal measured upon TX-100 addition.

### 2.7. Cell Viability

PC12 cells established from a rat pheochromocytoma were obtained from the American Type Culture Collection (ATCC, Manassas, VA, USA). PC12 cells were maintained in DMEM-Glutamax supplemented with 100 IU/mL of penicillin, 100 µg/mL of streptomycin, 15% fetal horse serum, and 2.5% fetal bovine serum at 37 °C in a humidified atmosphere of 5% CO_2_. Prior to the cell viability assay, α-synuclein was incubated at 200 µM for 2 days. The cells were subcultured in 96-well culture plates (30 × 10^3^ cells/well) for 24 h and then treated with 500 nM of aggregated α-synuclein, with or without the further addition of stilbenes at concentrations ranging from 5 to 30 µM, for 24 h, in a serum-free culture medium. Stilbenes were dissolved in DMSO at a final concentration of 0.1%, which is a subtoxic concentration. Cell viability was determined by using the MTT reduction assay. PC12 cells were incubated in 0.5 mg/mL of MTT at 37 °C for 3 h. Then, the MTT solution was removed, and the resulting formazan crystals were dissolved with DMSO. Absorbance values were read at 540 nm on a microplate reader (Dynex, Chantilly, VA, USA). All samples were analyzed in triplicate.

### 2.8. Statistical Analysis

All samples were analyzed at least in triplicate. Data are expressed as means ± standard errors. Statistical tests for PC12 cell experiments were performed with one-way ANOVA followed by Dunnett’s multiple comparison *post-hoc* test. Significance was set at *p* < 0.05. These analyses were performed with GraphPad Prism 5.03 for Windows (GraphPad Software, San Diego, CA, USA).

## 3. Results

### 3.1. Inhibition of α-Synuclein Fibril Formation

**Figure 1:**
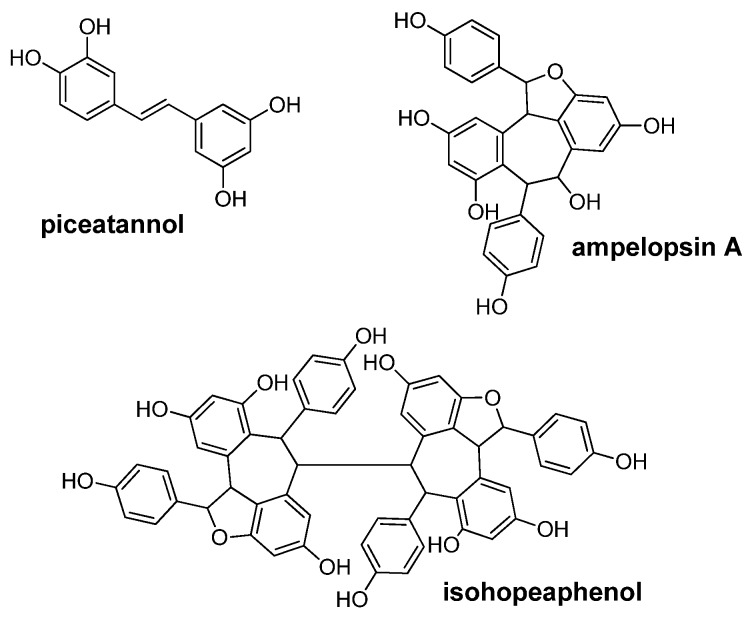
The structure of stilbenes.

Finding molecules to prevent the aggregation of α-synuclein could be a therapeutic goal in PD and related diseases [11,12]. Three stilbenes (Figure 1)—a monomer (piceatannol), a dimer (ampelopsin A), and a tetramer (isohopeaphenol)—were tested for their capacity to inhibit α-synuclein fibril formation.

**Figure 2:**
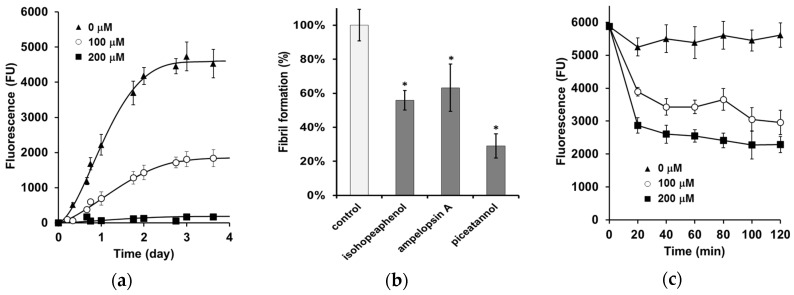
Inhibition and destabilization of α-synuclein fibrils by stilbenes: (**a**) α-synuclein (70 µM) was incubated for 4 days at 37 °C in a 20 mM Na_2_HPO_4_/NaH_2_PO_4_ 140 mM Tris buffer, pH 7.4 in a 96-well plate with piceatannol (0, 100, and 200 µM); (**b**) stilbene screening at 100 µM; (**c**) piceatannol (0, 100, and 200 µM) was added to previously aggregated fibrils in the aforementioned conditions for 4 days. Data are expressed as mean ± SD of three independent experiments. Results are expressed as mean ± SD* *p* < 0.05 *versus* control alone.

To determine whether stilbenes inhibit the assembly of α-synuclein into filaments, thT fluorescence was used in the presence or absence of stilbenes. thT fluorescence is correlated to β-sheet formation and to fibril formation [29]. The level of thT fluorescence was used to quantify filaments in the presence of each stilbene. In the absence of phenolic compounds, α-synuclein exhibits a quasi-sigmoidal binding curve with a lag phase of half a day, a period of increasing thT binding for three days, and then a binding plateau after three days (Figure 2a). These results are in agreement with the nucleation-dependent polymerization model of α-synuclein [30]. To evaluate the inhibitory capacity of stilbenes, initial screening for inhibition was performed at a concentration of 100 µM of each compound (Figure 2b). The results were expressed as the percentage of α-synuclein assembly in the absence of compound (taken to be 100%). All three compounds inhibited α-synuclein fibril formation, but the oligomers were less active than piceatannol (aggregation reduced to 29%). When α-synuclein was incubated with piceatannol, significant concentration-dependent effects were observed (Figure 2a). The lag time increased, the β-sheet growth rates decreased, and the final equilibrium levels decreased.

**Figure 3:**
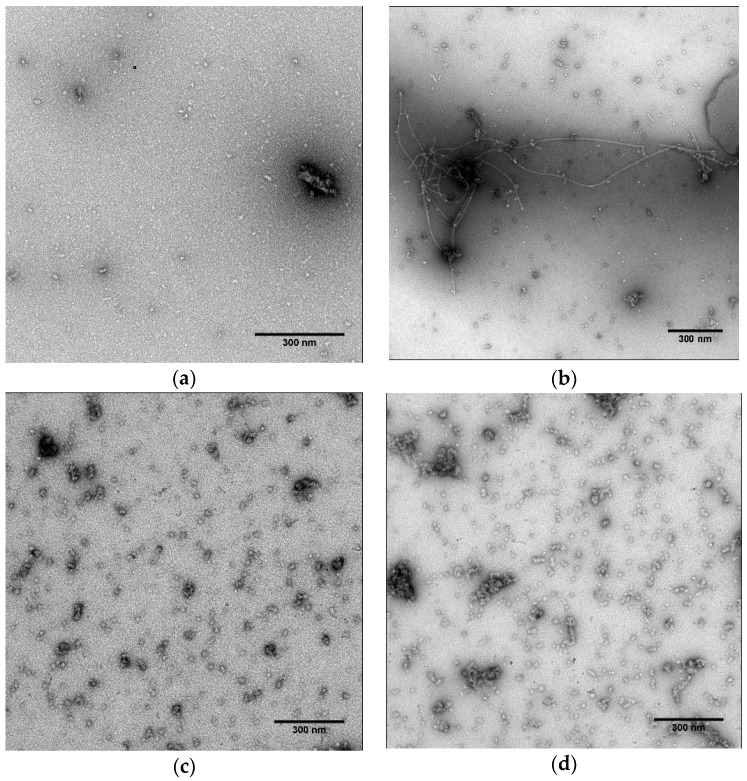
Direct transmission electron microscopy (TEM) observation of α-synuclein aggregation states: (**a**) α-synuclein (70 µM in a 20 mM Na_2_HPO_4_/NaH_2_PO_4_ 140 mM Tris buffer, pH 7.4) before aggregation; (**b**) aggregated alone for 4 days at 37 °C; or (**c**) with 100 µM of piceatannol. To observe fibril destabilization; (**d**) 100 µM of piceatannol was added to α-synuclein fibrils and then observed after 15 min. Scale bar indicates 300 nm.

To observe the morphology of α-synuclein aggregates, electron microscopic studies were performed. Before incubation, only small amorphous aggregates were observed in a sample of untreated α-synuclein (Figure 3a). After incubation of α-synuclein alone for 4 days at 37 °C, clear classical α-synuclein fibril extensions were observed (Figure 3b). The fibrils were composed of helical filaments 20 nm in diameter, as previously reported [29]. Efficient inhibition of α-synuclein was obtained after the addition of 100 µM of piceatannol (Figure 3c). Only small amorphous aggregates were observed.

**Figure 4:**
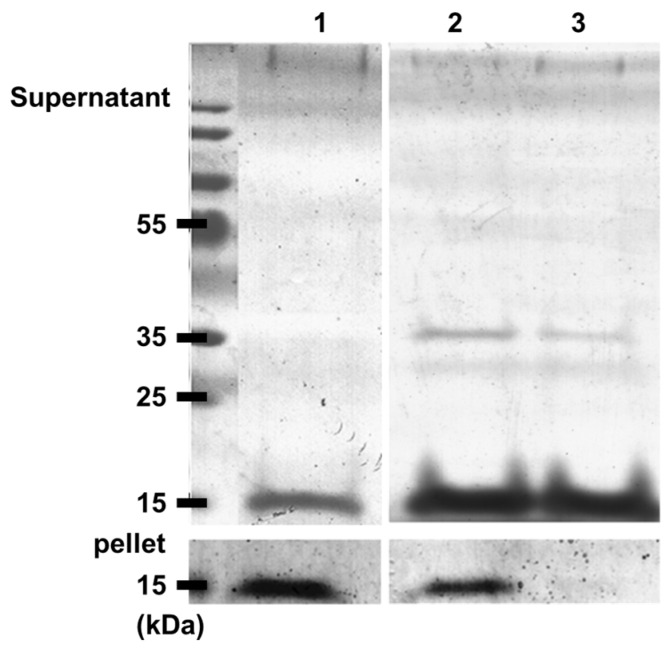
α-synuclein oligomerization followed by SDS-PAGE and Coomassie Blue staining. Centrifugation and analysis of proteins in supernatant and pellet fractions by SDS-PAGE were performed in absence and in presence of piceatannol. Lane 1, proteins alone (70 µM); Lanes 2 and 3, proteins plus piceatannol (100 and 200 µM, respectively).

To determine whether inhibition of α-synuclein fibril formation induces the formation of small stable α-synuclein oligomers, centrifugation and analysis of proteins in supernatant and pellet fractions by SDS-PAGE were performed in the absence and presence of piceatannol (Figure 4). α-synuclein incubated for 4 days at 37 °C alone exhibited a strong band in the pellet fraction and a small amount of soluble α-synuclein species in the supernatant fraction. Therefore, most of the protein was insoluble. In the presence of stilbene monomer, SDS-PAGE showed that the α-synuclein concentration in the pellet decreased, thereby confirming the loss of insoluble α-synuclein species. Moreover, weak bands corresponding to monomers or small oligomers were observed.

### 3.2. α-Synuclein Fibril Destabilization

To determine whether stilbenes can destabilize α-synuclein fibrils, the thT fluorescence signal of 70 µM of α-synuclein previously incubated for 96 h at 37 °C was monitored with and without the addition of piceatannol. In the absence of stilbene, the signal was almost unmodified for 24 h as previously reported [31]. In contrast, incubation of α-synuclein fibrils with piceatannol (Figure 2c) induced a strong and rapid decrease in the signal in a concentration-dependent manner. Half an hour after the addition of piceatannol, the signal reached a plateau, which suggests a decrease in α-synuclein fibril levels in solution.

TEM studies were performed to observe the morphologic changes of α-synuclein fibrils after the addition of piceatannol. Before addition, classical α-synuclein fibrils were observed as previously mentioned. Fifteen minutes after the addition of 100 µM of piceatannol on α-synuclein fibrils, the number of fibrils was efficiently reduced, and only small amorphous aggregates remained (Figure 3d).

### 3.3. The Inhibition of α-Synuclein-Induced Membrane Permeability

**Figure 5:**
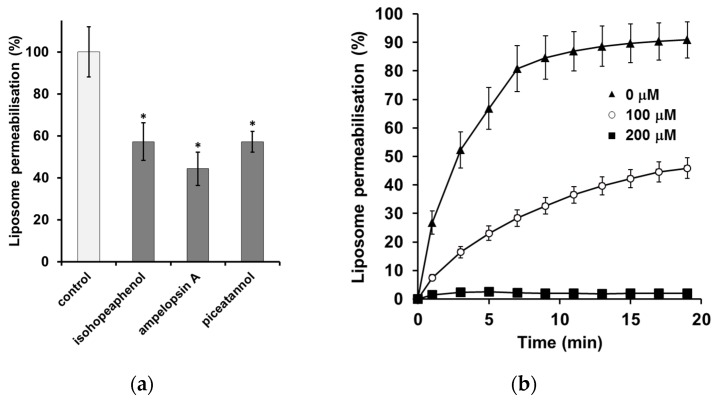
Effect of incubation with stilbenes on α-synuclein-induced content leakage from phosphatidyl inositol LUVs: (**a**) Stilbenes were each incubated at 100 µM with α synuclein. Leakage is expressed as a percentage of the effect induced by the addition of α synuclein aggregated alone; (**b**) α-synuclein was incubated with 0 µM, 100 µM, or 200 µM of piceatannol. Leakage was recorded for 20 min, and results are expressed as a percentage of total calcein leak induced by the addition of Triton X-100. The phospholipidic solution was 20 µM. α-synuclein was aggregated at 70 µM alone or with stilbenes for 6 days at 37 °C in a 20 mM Na_2_HPO_4_/NaH_2_PO_4_ 140 mM Tris buffer, pH 7.4 prior to the experiment. Data are expressed as mean ± SD of 3 independent experiments. * *p* < 0.05 *versus* control alone.

The ability of α-synuclein to disrupt lipidic membranes was monitored using a dye release assay. Calcein, a self-quenching fluorescent dye, was encapsulated in the vesicle. Aggregated α-synuclein (6 days at 37 °C) with or without stilbenes was incubated for 20 min with LUVs loaded with calcein. The increase in fluorescence intensity due to the release of calcein from the vesicle was observed. Aggregated α-synuclein led to a release of dye from the LUVs, indicating that protofibrils and fibrils of α-synuclein induce membrane disruption (Figure 5a). To determine the inhibitory capacity of stilbenes against aggregated α-synuclein-induced membrane disruption, initial screening was performed at a concentration of 100 µM of each compound (Figure 5a). All three compounds inhibited α-synuclein-induced membrane disruption. When α-synuclein was incubated with piceatannol, a significant concentration-dependent effect was observed (Figure 5b).

### 3.4. α-Synuclein-Mediated Cellular Toxicity

To study the ability of stilbenes to block α-synuclein-mediated cellular toxicity, an MTT assay was performed using PC12 line cells. MTT is a tetrazolium salt reduced to formazan by mitochondrial dehydrogenase only in living functional cells. First, the cytotoxic potential of each stilbene on PC12 cells was measured. Ampelopsin A and piceatannol are not cytotoxic at concentrations below 30 µM, while isohopeaphenol reduces cell viability at 10 µM and 30 µM (data not shown).

**Figure 6:**
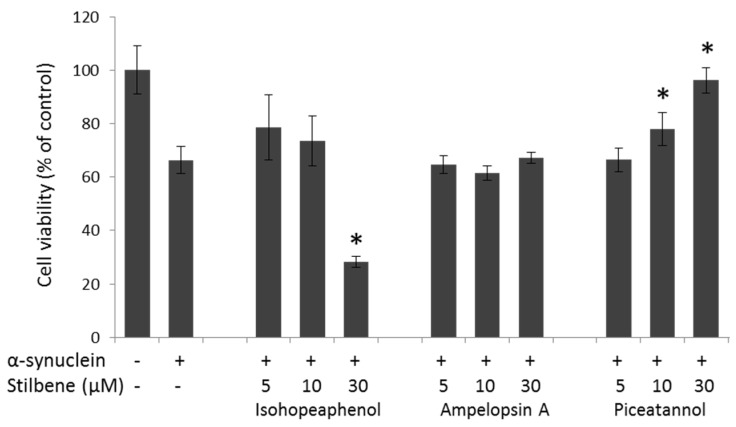
Cytotoxicity of α-synuclein aggregates. Cell viability was determined by using the MTT reduction assay. Results are expressed as the percentage of cell viability compared with the untreated control cells. The addition of 500 nM of aggregated α-synuclein alone on PC12 cells caused a significant loss of viability. Effects of stilbenes on cell viability were studied after the addition of 5 µM, 10 µM, and 30 µM of isohopeaphenol, ampelopsin A, and piceatannol. Results are expressed as mean ± SD * *p* < 0.05 *versus* α-synuclein alone.

When 500 nM of aggregated α-synuclein (dilution from 200 µM of α-synuclein incubated for 48 h at 37 °C) were added to PC12, cell viability was reduced to 65% of that of the control (Figure 6). Stilbenes at 5, 10, and 30 µM were added to PC12 cells simultaneously to α-synuclein for 24 h of treatment. Ampelopsin A had no protective effect against α-synuclein-induced toxicity (Figure 6). Isohopeaphenol had a non-significant protective effect at 5 and 10 µM and reduced cell viability at 30 µM (Figure 6). Piceatannol protected cells against α-synuclein-induced toxicity from 10 µM and restored cell viability at 30 µM (Figure 6). These results indicate that piceatannol attenuates α-synuclein-induced cytotoxicity on neuronal-like cells.

## 4. Discussion

α-synuclein is associated with pathological lesions in neurodegenerative diseases such as PD and some other related disorders [4]. Convergent evidence suggests that the assembly of α-synuclein plays a crucial role in the development of Lewy body diseases [5,6]. Therefore, the identification of compounds that can prevent or reverse the aggregation process could be a therapeutic strategy against these neurodegenerative disorders. Evidence is accumulating that small molecule inhibitors such as phenolic compounds could prevent α-synuclein aggregation and cytotoxicity [13,14,15,16,17,31,32].

Stilbenes are widely found in the plant kingdom, grapes and related products being their most significant dietary source [22]. The stilbene structure is derived from that of resveratrol. A substantial body of evidence suggests that resveratrol could have a beneficial effect on human health through a number of mechanisms [33]. Many studies have shown that resveratrol has a protective effect against neurodegenerative pathologies [34,35,36,37,38]. Resveratrol inhibits fibril formation by a variety of amyloidogenic proteins [13,37]. Recently, we identified resveratrol dimers that inhibit β-amyloid aggregation [23] and protect PC12 cells against β-amyloid-induced cytotoxicity [25]. In this study, we sought to evaluate the inhibitory properties of other wine stilbenes against α-synuclein fibril formation and cytotoxicity.

thT fluorescence, electronic microscopy, and SDS-PAGE analysis were performed to investigate the effects of three stilbenes on α-synuclein filament formation and destabilization. In order to investigate the effects of the different classes of stilbenes, a monomer (piceatannol), a dimer (ampelopsin A), and a tetramer (isohopeaphenol) were investigated. All stilbenes were extracted from vine stalks. Initial screening by thT fluorescence assays was performed to compare the anti-assembly effects of stilbenes at a concentration of 100 µM. Piceatannol exhibited the strongest inhibitory activity against α-synuclein aggregation, while ampelopsin A and hopeaphenol were less active. The inhibitory capacity of piceatannol against the formation of α-synuclein filaments was confirmed by TEM and SDS-PAGE analysis. TEM images showed that α-synuclein in the presence of piceatannol only forms small amorphous aggregates. Furthermore, thT fluorescence and TEM analysis indicated that this monomer is able to disaggregate preformed α-synuclein filaments. These findings are consistent with literature suggesting that phenolic compounds with adjacent hydroxyl groups on the same ring hinder the progress of the self-assembly process more efficiently [29]. Our results are in agreement with this observation. Stilbene oligomers were less active. These findings indicate that spatial constraints are critical in the inhibitory process. Nevertheless, additional experiments are needed to confirm and understand these structure-activity relationships.

The α-synuclein oligomers induce membrane damage, which implies a pore-like form of permeabilization [7,39]. Hence, identifying compounds that can prevent the disruption and permeabilization of membranes could be of therapeutic value. All stilbenes inhibit the liposome permeabilization induced by aggregated α-synuclein. These findings are in agreement with previous results suggesting that polyphenols can inhibit the membrane disruption induced by α-synuclein oligomers [19]. Altogether, these data suggest that stilbenes could induce the formation of soluble species that can prevent the disruption and permeabilization of membranes induced by aggregated α-synuclein alone.

Finally, cell viability was tested on rat pheochromocytoma neuronal-like cells (PC12) to assess the toxicity of α-synuclein and the preventive effects of stilbenes [40]. As previously reported, aggregated α-synuclein was found to reduce PC12 cell viability. Stilbene oligomers did not prevent α-synuclein-induced cytotoxicity. In contrast, dose-dependency experiments showed that piceatannol has a preventive effect. These findings indicate that piceatannol inhibits not only α-synuclein fibril formation but also α-synuclein-induced cytotoxicity.

This study demonstrates that piceatannol could inhibit α-synuclein fibrillation and toxicity by forming soluble non-toxic α-synuclein/polyphenol oligomers. While piceatannol occurs naturally in various plants, its bioavailability is still practically unknown. Piceatannol, compared to resveratrol, seems to possess more moderate oral bioavailability when fed to rats [41]. In addition, *in vitro* and *in vivo* studies indicate that resveratrol could be metabolized into piceatannol [42]. Piceatannol as resveratrol can dock to transportation proteins such as transthyretin, suggesting its possible transport in the serum and cerebrospinal fluid [43]. Resveratrol can cross the blood*–*brain barrier and exhibit neuroprotective properties against cerebral injury [44]. Shu *et al.* reported the bioavailability of resveratrol administered to rat brains by different routes such as intragastric ingestion, intraperitoneal injection, external carotid artery injection, and lumbar puncture injection [45]. They observed that resveratrol could reach concentrations around 10 nmol/g in different parts of the brain after a lumbar puncture injection of 50 nmol of resveratrol. Concerning another stilbene monomers, it had been demonstrated that the oral administration of pterostilbene exerts a neuroprotective effect against acute cerebral injury in mice [46]. This compound is able to pass through the blood*–*brain barrier. Taking into account these results, it is reasonable to expect that it is possible to lead piceatannol directly into the brain to develop therapeutic treatments.

## Abbreviations

The following abbreviations are used in this manuscript:

LUVs	large unilamellar vesicles
MTT	3-(4,5-dimethylthiazol-2-yl)-2,5-diphenyltetrazolium bromide
thT	thioflavin T
PD	Parkinson’s disease
